# 5 challenges in understanding the role of the virome in health and disease

**DOI:** 10.1371/journal.ppat.1008318

**Published:** 2020-03-26

**Authors:** David Wang

**Affiliations:** Departments of Molecular Microbiology and Pathology and Immunology, Washington University in St. Louis School of Medicine, St. Louis, Missouri, United States of America; University of Michigan Medical School, UNITED STATES

Over the past decade, many studies have established linkages between the microbiome and states of health and disease. By contrast, understanding of the corresponding virome (i.e., the set of all viruses, both eukaryotic and prokaryotic, in a given niche) has lagged substantially behind. There are relatively few virome studies in comparison to microbiome studies. Nonetheless, in recent years, there has been an increasing recognition of the importance of the virome as it has been associated with diseases such as HIV and SIV infection [1, 2], inflammatory bowel disease [3], malnutrition [4], graft-versus-host disease [5], and type 1 diabetes [6, 7]. The identification of virome associations with disease is reminiscent of the descriptive studies that emerged in the early days of the bacterial microbiome. As was the case then, the most critical challenge ahead is defining whether the virome plays a causal role in the associated diseases. In addition, there are additional, unique challenges inherent to virome analysis that render it less tractable than the bacterial microbiome.

## Inability to identify all viruses due to the absence of a universal viral sequence: The challenge of viral “dark matter”

Comprehensive census of the bacterial and fungal microbiome can be achieved through consensus PCR approaches that target the 16S rRNA and internal transcribed spacer (ITS) loci, respectively. By contrast, there is no such analogous conserved sequence present in all viruses. The lack of a consensus sequence poses a significant challenge for efforts to systematically define the set of viruses present in a given specimen. Rather than simply amplifying and sequencing a signature target locus, metagenomic sequencing of nucleic acid in a sample is required for virus sequences to be represented in a sequencing library. The increased sequencing depth necessary dramatically increases the cost compared to 16S or ITS sequencing. Moreover, the relative abundance of viral to nonviral nucleic acids is an important parameter that drives sensitivity. To mitigate this problem, physical enrichment for viral particles by filtration and/or nuclease treatment is often necessary. Another consequence of the requirement for metagenomic sequencing is that the subsequent bioinformatic analysis is also much more complex; the metagenomic sequences must be aligned at both nucleotide and amino acid levels to large reference databases of viral sequences (not just to a database of reference amplicon nucleic acid sequences). Thus, the experimental sequencing depth, computational infrastructure requirements, and computing costs are significantly higher for virome analyses. The precise experimental and bioinformatic analysis steps utilized can contribute significantly to variability and, in some instances, potentially incorrect conclusions. However, a detailed discussion of both of these is beyond the scope of this review.

Currently, classification of sequence reads as being viral in origin relies primarily upon detectable primary sequence alignment to reference viruses. One challenge is that, among those that are alignable to reference viruses, sequences can be misclassified due to the remoteness of the similarity and/or low stringency. An even more significant problem is that in most virome studies, more than 50% of the sequences in virus-enriched preparations have no detectable sequence similarity to any known reference sequences; these unalignable sequences are referred to as viral “dark matter” [8] and may include novel, highly divergent viruses that are unrecognizable. Thus, most current virome studies are presenting only a partial view of the virome because the dark matter sequences are typically ignored. The dark matter clearly harbors hidden treasures because mining of the dark matter has led to discoveries such as crAssphage, the most abundant bacteriophage in human enteric viromes [9].

To computationally address the dark matter, new and improved data mining strategies are needed. One approach is to apply methods superior at detection of remote homologies, such as hidden Markov models customized for viral proteins [10]. Other machine learning strategies may include development of primary sequence-alignment–independent classification approaches [11] or development of artificial neural networks [12]. Experimentally, as described below, expanding the set of culturable viruses will provide additional reference sequences that should also decrease the amount of viral dark matter.

## Inadequate sampling strategies bias towards DNA viruses and against RNA viruses

One bias in many virome studies to date is a focus only on sequencing of DNA. This is due in part to the bacterial microbiome being the primary driver of many studies, and thus at the time of sampling, experimental measures to extract DNA were employed without regard to trying to either preserve or recover the RNA. In addition, some studies utilize DNA-specific amplification strategies such as phi29-mediated whole-genome amplification. These studies are unable to assess the RNA component of the virome. Yet the majority of the known eukaryotic viral denizens of the human enteric tract are RNA viruses, such as enteroviruses, rotaviruses, noroviruses, and astroviruses. On the phage side, while it is true that the vast majority of known phages have DNA genomes, the bias of sequencing exclusively DNA has served as a self-reinforcing positive feedback loop; however, recent studies examining samples that have sequenced RNA have demonstrated that there is a much greater abundance and diversity of RNA phages in the world [13]. Thus, in order achieve a more complete view of the virome, it is critical that virome studies be designed such that RNA viruses are preserved in the specimens and that the RNA fraction is incorporated into the sequencing and analysis.

## Lack of culture systems to propagate components of the virome

The advent of metagenomics has greatly enhanced our ability to detect known and novel viral sequences in unbiased fashion and to establish novel associations of these sequences with various disease [1–7]. However, it is not known whether the virome plays a causative role or not. Koch’s postulates remain the gold standard for microbial disease causality, and thus the first step is to establish culture systems for viruses associated with the disease of interest. The lack of culture systems for viruses identified in virome studies, for both eukaryotic viruses and phages, is pronounced. As an example, although dozens of novel eukaryotic viruses have been identified in the mammalian enteric tract by metagenomic sequencing, culture systems for only a very limited number have been described to date [14, 15]. Likewise, for phages, genomic sequences of thousands of novel phages can be identified in a single study [16, 17], but very few have been isolated [18]. Thus, while (in the past) discovery of novel viruses was rate limiting, today the rate-limiting step has shifted to development of culture systems for the viruses that have been molecularly identified.

How can this be addressed? To some extent, this is simply a matter of effort. In all likelihood, no effort has been made to culture the vast majority of the novel viruses identified in virome studies, and some will surely succumb to standard culture conditions once applied. Limitations in quantity or quality of primary specimens containing viable virus can contribute to the problem. In part, it is challenging for labs to obtain funding to culture novel viruses, particularly in the absence of any strong disease association. Moreover, the risk of a negative result, exemplified by many decades of unsuccessful attempts to culture human norovirus, is substantial. Nonetheless, given that the lack of ability to culture a virus is perhaps the most fundamental barrier to progress in the study of that virus, dedicated efforts to develop culture systems are absolutely necessary.

For some of the more recently discovered eukaryotic viruses, the advent of primary, differentiated culture systems has been key to unlocking this riddle. For example, the respiratory viruses bocavirus and coronavirus HKU1 can be propagated using primary airway epithelial cells [19, 20]. In the enteric tract, the development of enteroids and organoids and recognition of the need for additional enteric tract components have opened the frontier for propagation of human norovirus [21, 22]. Broader application of these systems will undoubtedly lead to increased success in culture of some eukaryotic viruses. On the phage front, one significant challenge is that many of the bacterial host species are themselves unculturable using standard bacterial-growth media. Thus, efforts to propagate novel phages will also entail improving systems for bacterial in vitro growth. Some approaches toward this include development of bioreactors [23, 24] and other systems that better mimic the natural environment [25], but additional new and innovative strategies are needed.

## The need for experimental animal-infection models

For the majority of complex diseases, in vitro systems are not adequate to assess viral contributions to pathogenicity, and thus appropriate animal-disease models are required that also reflect the potentially complex interactions with the host, bacteria, and eukaryotic microbes that may be present. In bacterial microbiome studies, a powerful approach has been the ability to colonize mice with either a single defined bacteria of interest or a consortia of bacteria. By analogy, it would be ideal if the virome could be functionally interrogated in similar fashion. However, a prerequisite for eukaryotic viruses is that one must first be able to establish infection in the relevant animal model. As with establishing cell-culture systems, the paucity of animal-infection models for novel viruses stems in part from lack of effort to develop such systems. Moreover, the challenge is exacerbated by the multitude of potential infection routes that need to be evaluated, need to overcome the immune response, cross-species transmission barriers, and (even within a species) potential impact of varying genetic backgrounds. Use of transgenic animals that are immunodeficient [26] or that have been partially humanized [27] has enabled development of murine models for some viruses. In an ideal world, animal models for all eukaryotic viruses identified in virome studies would be established, enabling the roles of any single virus (or cocktail of viruses) to be functionally evaluated. Likewise, having an extensive library of cultivated phages (see above) will allow them to be combined in defined proportions along with eukaryotic viruses for experimental studies. For phages, presuming they act by modulating the bacterial community, the animal model must harbor the relevant bacterial host species, and thus the bacterial microbiome may need to be manipulated as well. Notably, some recent studies have suggested that phages may also interact directly with eukaryotic host cells [28, 29]. Overall, significant effort and resources must be expended to establish robust animal-infection models suitable to define the role of the virome. This is a step that is absolutely necessary in order to move virome studies beyond the realm of mere association studies.

## Dichotomy between eukaryotic virus and phage communities

A barrier to progress in the virome field is the division between virologists who study viruses infecting eukaryotes versus those that infect bacteria. Very few scientists today have expertise in both. This balkanization of virology leads to challenges when an eukaryotic virologist discovers in a virome study that the strongest disease association is with a phage (or vice versa). Lack of familiarity with the field and relevant experimental approaches often limits further investigation. These communities have been largely segregated, often holding separate conferences and distinct grant-review panels. To illustrate this point, the United States National Institutes of Health (NIH) virology study sections address only “non-bacteriophage viral genetics, infection and replication, cellular and host responses to viral infections, and mechanisms of viral disease pathogenesis.” Thus, there is a great need to bring these disparate communities together in order to collectively attack questions associated with the virome, especially as more complex trans-kingdom interactions are identified linking phages, bacteria, eukaryotic viruses, and eukaryotic cells.

In conclusion, the coming years will undoubtedly be witness to many more studies demonstrating associations of the virome with various diseases. Hopefully, there will be commensurate development of new computational approaches that significantly decrease the fraction of viral dark matter and an increase in the fraction of studies that holistically evaluate the virome. With new cell-culture systems and animal models for novel viruses, there will ideally be studies that attribute causal roles for some of the associations. Finally, it may be that virome studies will serve as a catalyst to help integrate the eukaryotic viral and phage communities.
